# Brain microRNAs are associated with variation in cognitive trajectory in advanced age

**DOI:** 10.1038/s41398-022-01806-3

**Published:** 2022-02-01

**Authors:** Aliza P. Wingo, Mengli Wang, Jiaqi Liu, Michael S. Breen, Hyun-Sik Yang, Beisha Tang, Julie A. Schneider, Nicholas T. Seyfried, James J. Lah, Allan I. Levey, David A. Bennett, Peng Jin, Philip L. De Jager, Thomas S. Wingo

**Affiliations:** 1 Division of Mental Health, Atlanta VA Medical Center, Decatur, GA USA; 2 Department of Psychiatry and Behavioral Sciences, Emory University School of Medicine, Atlanta, GA USA; 3 Department of Neurology, Xiangya Hospital, Central South University, Changsha, Hunan China; 4 Department of Neurology, Emory University School of Medicine, Atlanta, GA USA; 5 Department of Psychiatry, Icahn School of Medicine at Mount Sinai, New York, NY USA; 6 Department of Genetics and Genomic Sciences, Icahn School of Medicine at Mount Sinai, New York, NY USA; 7 Seaver Autism Center for Research and Treatment, Icahn School of Medicine at Mount Sinai, New York, NY USA; 8 Center for Alzheimer Research and Treatment, Brigham and Women’s Hospital, Harvard Medical School, Boston, MA USA; 9 Cell Circuits Program, Broad Institute, Cambridge, MA USA; 10 National Clinical Research Center for Geriatric Disorders (XIANGYA), Changsha, Hunan China; 11 Rush Alzheimer’s Disease Center, Rush University Medical Center, Chicago, IL USA; 12 Department of Biochemistry, Emory University School of Medicine, Atlanta, GA USA; 13 Department of Human Genetics, Emory University School of Medicine, Atlanta, GA USA; 14 Center for Translational and Computational Neuroimmunology, Department of Neurology, Columbia University Medical Center, New York, NY USA

**Keywords:** Molecular neuroscience, Epigenetics in the nervous system, Pathogenesis

## Abstract

In advancing age, some individuals maintain a stable cognitive performance over time, while others experience a rapid decline. Such variation in cognitive trajectory is only partially explained by common neurodegenerative pathologies. Hence, we aimed to identify new molecular processes underlying variation in cognitive trajectory using brain microRNA profile followed by an integrative analysis with brain transcriptome and proteome. Individual cognitive trajectories were derived from longitudinally assessed cognitive-test scores of older-adult brain donors from four longitudinal cohorts. Postmortem brain microRNA profiles, transcriptomes, and proteomes were derived from the dorsolateral prefrontal cortex. The global microRNA association study of cognitive trajectory was performed in a discovery (*n* = 454) and replication cohort (*n* = 134), followed by a meta-analysis that identified 6 microRNAs. Among these, miR-132-3p and miR-29a-3p were most significantly associated with cognitive trajectory. They explain 18.2% and 2.0% of the variance of cognitive trajectory, respectively, and act independently of the eight measured neurodegenerative pathologies. Furthermore, integrative transcriptomic and proteomic analyses revealed that miR-132-3p was significantly associated with 24 of the 47 modules of co-expressed genes of the transcriptome, miR-29a-3p with 3 modules, and identified 84 and 214 downstream targets of miR-132-3p and miR-29a-3p, respectively, in cognitive trajectory. This is the first global microRNA study of cognitive trajectory to our knowledge. We identified miR-29a-3p and miR-132-3p as novel and robust contributors to cognitive trajectory independently of the eight known cerebral pathologies. Our findings lay a foundation for future studies investigating mechanisms and developing interventions to enhance cognitive stability in advanced age.

In advancing age, the trajectory of cognitive performance over time can range from stability to rapid decline. While cognitive decline, especially rapid decline, may ultimately lead to a diagnosis of mild cognitive impairment (MCI) or dementia, cognitive stability over time may reflect cognitive resilience. Hence, variation in cognitive trajectory can influence dementia risk, as well as the age of onset for MCI or dementia. Studying individual cognitive trajectories is strategic for several reasons. First, it captures all the factors influencing cognitive performance including diverse pathologies and biological mechanisms independent of pathologies [1–3]. Second, it likely captures co-occurring disease processes and co-occurring age-related pathologies which are known to be prevalent in the brains of aged individuals [1, 2]. Notably, variation in individual cognitive trajectory is only partially explained by traditional neurodegenerative pathologies [2, 4]. In particular, β-amyloid plaques, neurofibrillary tangles, microinfarct, macroinfarct, and Lewy bodies together capture only about 40% of the variance in cognitive trajectory, leaving 60% unexplained [2, 4]. This suggests that mechanisms other than pathologies can contribute to individual differences in cognitive trajectory.

microRNAs (miRNAs) are small non-coding RNAs that exert a crucial layer of post-transcriptional regulation on gene expression by either degrading the target mRNAs or repressing the translation of mRNAs into proteins [5]. miRNAs are sometimes referred to as “master regulators” because one miRNA can regulate up to hundreds of mRNAs to exert substantial effects on gene expression networks [6, 7]. miRNAs have been shown to be important for synaptic plasticity, aggregation of neurodegenerative pathologies, neuronal survival, learning, memory consolidation, and memory retrieval [8–11]. In the cortical data analyzed here, we previously found associations between miRNAs and the pathologies that define Alzheimer’s disease (β-amyloid and tau) [12]. Hence, we hypothesized that miRNAs are also important for cognitive trajectory in advanced age.

Here we aimed to identify new biological processes underlying variation in cognitive trajectory by investigating the role of miRNAs and their downstream target mRNAs and proteins in cognitive trajectory in four longitudinal cohorts of older adults. We first performed a global miRNA association study of cognitive trajectory in a discovery and replication cohort, followed by a meta-analysis (Fig. 1). Then we performed integrative analyses between the significant miRNAs and the transcriptome and proteome, all of which were generated from the dorsolateral prefrontal cortex (Fig. 1). Together our multi-omics analyses provide a new framework for understanding the roles of miRNAs in cognitive trajectory.

**Fig. 1:**
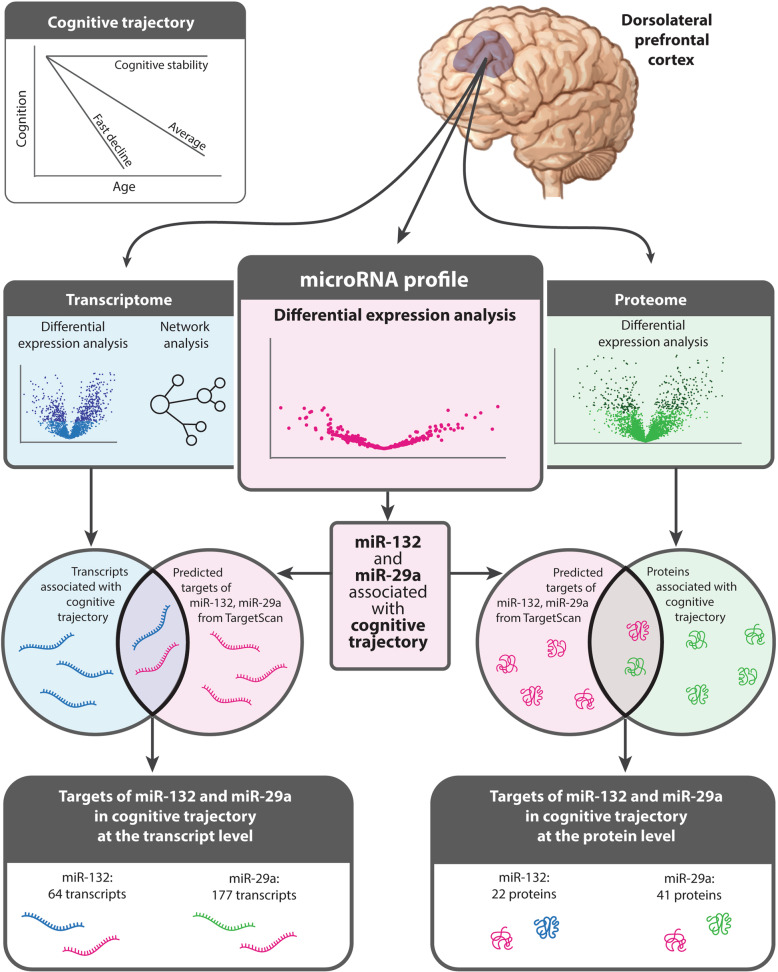
Overview of the study design and findings. Individual cognitive trajectories were estimated from annual cognitive testing scores in participants of the ROS/MAP, Banner, and BLSA cohorts, respectively. These participants donated their brains at death. In ROS/MAP, global microRNAs and transcriptomes were profiled from the dorsolateral prefrontal cortex (dlPFC). Likewise, in Banner and BLSA, proteomes were profiled from the dlPFC. A global miRNA association study of cognitive trajectory was performed followed by a transcriptome-wide and proteome-wide association studies of cognitive trajectory. Next, an integrative analysis was performed to identify downstream targets of the cognitive trajectory-associated miRNAs at the transcript and protein levels.

## Materials and methods

A detailed description is found in supplement methods.

### Study design and participants

Participants for the discovery and replication cohorts for the miRNA analysis were from two longitudinal clinical-pathologic cohort studies of aging and Alzheimer’s disease—the Religious Orders Study (ROS) and Rush Memory and Aging Project (MAP) [13]. Both studies involve detailed annual cognitive and clinical evaluations and brain autopsy. To be included in our miRNA study, participants must have at least one follow-up evaluation and did not have a diagnosis of dementia at baseline. Participants with proteomic profiles were recruited by the Banner Sun Health Research Institute (Banner) [14] and Baltimore Longitudinal Study of Aging (BLSA) [15, 16] and were followed longitudinally and annually using standardized neurological and neuropsychological tests [14].

### Clinical traits and cerebral pathologies

*Cognitive trajectory* refers to the person-specific rate of change of global cognitive performance over time. Annually, 17 cognitive tests were administered to each ROS/MAP participant to assess episodic memory, perceptual orientation, perceptual speed, semantic memory, and working memory [17]. Rate of cognitive change is the random slope from a linear mixed-effects model in which the annual global cognitive performance was the longitudinal outcome, follow-up year as the independent variable, adjusting for age at recruitment, sex, and years of education [18–21]. Likewise, for Banner and BLSA participants, the person-specific cognitive trajectory was estimated using a linear mixed model. In this model, the annual Mini-Mental State Examination (MMSE) [22] score was the longitudinal outcome, follow-up year as the independent variable, sex, education, and age at follow-up year as the covariates, and with random intercept and random slope per subject.

#### Cerebral pathologies

We included eight measured cerebral pathologies in our ROS/MAP analyses. They are neurofibrillary tangles, β-amyloid, Lewy bodies, gross cerebral infarct, microinfarcts, cerebral atherosclerosis, cerebral amyloid angiopathy, and hippocampal sclerosis as described in detail in the supplementary methods.

*Clinical diagnosis of cognitive status* (control, mild cognitive impairment (MCI), or dementia) was rendered at every assessment based on a three-stage process, including cognitive-test scores, clinical judgment by a neuropsychologist, and diagnostic classification by a neurologist [23].

### microRNA quantification and quality control

Total RNA, including miRNA, was extracted from ROS/MAP postmortem brain tissue from the dorsolateral prefrontal cortex (dlPFC). miRNAs were profiled using the nCounter Human miRNA and described in detail previously [12]. We retained miRNAs with call rate ≥95% and with an absolute value of >15 in at least 50% of the samples [12]. We then removed batch effects using Combat [12, 24]. After quality control, a total of 292 miRNAs were included in the miRNA association study.

### Transcriptome profiling and quality control

RNA extracted from ROS/MAP postmortem dlPFC was sequenced on the Illumina HiSeq. Alignment of reads to GRCh38 reference genome was performed using STAR [25]. Gene level counts were computed. Genes with <1 count per million in at least 50% of the samples and with missing length and percent GC content were removed. After quality control, there were 15,582 genes to be included in the transcriptome-wide differential expression analysis of cognitive trajectory.

*Proportions of neurons, astrocytes, oligodendrocytes, and microglia* were estimated from RNA-sequencing data using CIBERSORT [26] and cell-type-specific signatures from Darmanis et al. [27]. We used the proportions of cell type to adjust for tissue heterogeneity in the global miRNA association study and transcriptome-wide differential expression analysis of cognitive trajectory.

### Proteome quantification and quality control

Whole-brain proteomes were derived from postmortem dlPFC of Banner and BLSA donors. Proteomic quantification for both cohorts was described previously [28]. Only proteins quantified in at least 90% of the samples (3710 proteins in Banner and 3933 proteins in BLSA cohorts) were included in the cognitive trajectory analysis. Within each cohort, protein abundance was transformed using log2, then batch effects were removed using Combat [24], and effects of age at death, sex, and postmortem interval (PMI) were removed using bootstrap regression as described previously [28].

### Validation of targets of miR-132-3p and miR-29a-3p

Putative targets of miR-132-3p and miR-29a-3p in cognitive trajectory were validated using luciferase reporter assays as described in detail in supplementary methods.

### Statistical analysis

A global microRNA association study of cognitive trajectory was performed in the discovery cohort using limma [29], adjusting for sex, age at death, RNA integrity number (RIN), postmortem interval (PMI), study (ROS versus MAP), and proportions of neuron, astrocyte, oligodendrocyte, and microglia. Likewise, a global miRNA association study of cognitive trajectory was performed in the replication cohort using the same approach except that the replication cohort lacks estimates of brain cell types. Meta-analysis of the findings from the discovery and replication cohort was performed with METAL using effect size estimates and standard errors [30]. For all analyses, multiple testing adjustment was addressed with Benjamini-Hochberg (BH) false discovery rate (FDR) [31].

*Pairwise correlation among the cognitive trajectory-associated miRNAs* was performed with Pearson correlation, adjusting for multiple testing. We regressed out effects of sex, age at death, RIN, PMI, study, and proportions of neuron, astrocyte, oligodendrocyte, and microglia from the miRNA profile to yield a normalized miRNA profile before performing the pairwise correlation.

#### Percent variance of cognitive trajectory

Likewise, we used the normalized miRNA profile as described above to estimate the percent variance of cognitive trajectory explained by particular miRNAs and each of the eight cerebral pathologies. Specifically, we used a fixed-effect model in *variancePartition* package to estimates the effect each of the assessed variables contributes to cognitive trajectory while correcting for the contribution of all the others [32]. This method considers all the variables jointly and provides a framework for comparing contribution of a particular miRNA to cognitive trajectory to that of each of the known pathologies.

#### Correlation between gene co-expression modules and cognitive trajectory-associated miRNAs

We used the normalized miRNA profile as described above to examine pair-wise correlations between cognitive trajectory-associated miRNAs and each of the 47 modules of co-expressed genes generated by Mostafavi et al [21] using Spearman correlation, adjusting for multiple testing. Each module was represented by the mean expression level of all the genes assigned to that module.

*Transcriptome-wide differential expression analysis of cognitive trajectory* was performed using voom-limma [29, 33] adjusting for sex, age at death, study, RIN, PMI, RNA-sequencing batch, proportions of neuron, astrocyte, oligodendrocyte, and microglia. Multiple testing adjustment was addressed with BH FDR [31].

*Proteome-wide association study (PWAS) of cognitive trajectory* was performed in Banner and BLSA, separately, followed by a meta-analysis and were done previously [28]. Briefly, in each cohort, a linear regression was performed with cognitive trajectory as the outcome and normalized protein abundance as the predictor. Of note, sex, age at death, and PMI have been regressed from the proteomic profile used in the PWAS. Likewise, sex, age, and education have been regressed during the derivation of cognitive trajectory. A meta-analysis was performed with METAL using effect size estimates and standard errors [30]. For all analyses, we used BH method to control the FDR [31]. We used the proteins found to be associated with cognitive trajectory at FDR < 0.05 from the meta-analysis for our integrative miRNA proteomic analysis to identify targets of miR-132-3p and miR-29a-3p in cognitive trajectory at the protein level.

## Results

### Study participants in the discovery and replication cohorts

There were 454 ROS/MAP participants in the discovery analysis and 134 ROS/MAP participants in the replication analysis (Table 1). The main distinguishing feature between the discovery and replication cohorts is that the former has transcriptomic profiles, while the latter does not. Both the discovery and replication cohorts were followed prospectively with annual cognitive evaluations for a median of 7 years and up to 16 years (Table 1). The median age at baseline was 81 and 80 years for the discovery and replication cohorts, respectively, and the median age at death was 89 and 87 years, respectively (Table 1). Median education was 16 years for both cohorts. Importantly, all participants did not have a diagnosis of dementia at baseline in both the discovery and replication cohorts. The final clinical diagnosis at the time of death consisted of 38% dementia, 28% MCI, 34% cognitively nonimpaired for the discovery set, and 49% dementia, 19% MCI, and 31% cognitively nonimpaired for the replication set (Table 1).

**Table 1:** Characteristics of the discovery and replication cohorts in the global microRNA association study of cognitive trajectory.

	ROS/MAP Discovery (*N* = 454)	ROS/MAP Replication (*N* = 134)
**Sex**		
Female	295 (65%)	94 (70%)
Male	159 (35%)	40 (30%)
**Age at baseline**		
Mean (SD)	81 (7.0)	80 (6.4)
Median (Min, Max)	81 (65, 102)	80 (65, 95)
**Age at death**		
Mean (SD)	89 (6.7)	87 (6.1)
Median (Min, Max)	89 (71, 108)	88 (70, 100)
**Follow-up duration**		
Mean (SD)	7.5 (3.75)	7.5 (3.74)
Median (Min, Max)	6.9 (0.775, 16.7)	7.0 (1.03, 16.1)
**Education**		
Mean (SD)	16.6 (3.44)	16.4 (3.79)
Median (Min, Max)	16.0 (5.00, 28.0)	16.0 (8.00, 25.0)
**Baseline cognitive diagnosis**		
Normal controls	285 (62.8%)	89 (66.4%)
MCI	169 (37.2%)	45 (33.6%)
Alzheimer’s disease	0 (0%)	0 (0%)
**Last cognitive diagnosis**		
Normal controls	154 (33.9%)	42 (31.3%)
MCI	129 (28.4%)	26 (19.4%)
Alzheimer’s disease	171 (37.7%)	66 (49.3%)
**Cognitive trajectory slope**		
Mean (SD)	−0.007 (0.089)	−0.044 (0.113)
Median (Min, Max)	0.012 (−0.361, 0.143)	−0.030 (−0.498, 0.149)
**RIN**		
Mean (SD)	7.2 (0.97)	4.5 (1.56)
Median (Min, Max)	7.4 (5.00, 9.90)	4.3 (1.00, 8.20)
**PMI**		
Mean (SD)	7.3 (4.95)	8.2 (8.38)
Median (Min, Max)	5.8 (0, 40.8)	6.0 (0, 85.1)
**Neurofibrillary tangles**		
Mean (SD)	5.5 (6.15)	6.8 (8.30)
Median (Min, Max)	3.7 (0, 38.8)	3.4 (0.00302, 61.0)
Missing	3 (0.7%)	1 (0.7%)
**B-amyloid**		
Mean (SD)	3.4 (3.70)	3.6 (3.95)
Median (Min, Max)	2.1 (0, 18.3)	2.2 (0, 19.1)
Missing	3 (0.7%)	1 (0.7%)
**Lewy bodies**		
not present	366 (80.6%)	106 (79.1%)
present	88 (19.4%)	28 (20.9%)
**Gross cerebral infarct**		
not present	257 (56.6%)	68 (50.7%)
present	197 (43.4%)	66 (49.3%)
**Microinfarcts**		
not present	340 (74.9%)	90 (67.2%)
present	114 (25.1%)	44 (32.8%)
**Cerebral atherosclerosis**		
none	46 (10.1%)	15 (11.2%)
mild	202 (44.5%)	55 (41.0%)
moderate	156 (34.4%)	47 (35.1%)
severe	48 (10.6%)	16 (11.9%)
Missing	2 (0.4%)	1 (0.7%)
**Cerebral amyloid angiopathy**		
none	101 (22.2%)	27 (20.1%)
mild	189 (41.6%)	57 (42.5%)
moderate	97 (21.4%)	32 (23.9%)
severe	58 (12.8%)	14 (10.4%)
Missing	9 (2.0%)	4 (3.0%)
**Hippocampal sclerosis**		
not present	423 (93.2%)	123 (91.8%)
present	27 (5.9%)	8 (6.0%)
Missing	4 (0.9%)	3 (2.2%)

### Global miRNA association study of cognitive trajectory identified six significant miRNAs

The cognitive trajectory for each subject was represented by its slope, which reflects the linear rate of change in cognitive performance over time, and was treated as a continuous variable (Table 1). Specifically, a trajectory with a positive slope or small negative slope indicates cognitive stability while a trajectory with a large negative slope reflects fast cognitive decline.

**Fig. 2:**
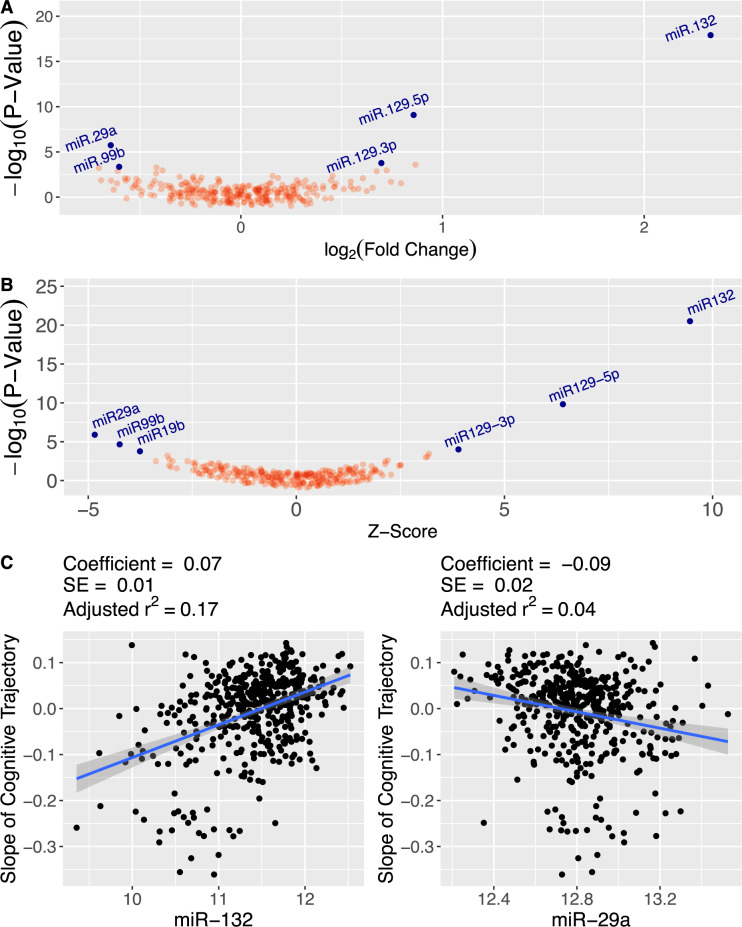
Global miRNA association study of cognitive trajectory in ROS/MAP. **A** Volcano plot of the global miRNA association study of cognitive trajectory in the discovery dataset. The miRNAs colored in blue are those associated with cognitive trajectory at adjusted *p* < 0.05. **B** Volcano plot for the meta-analysis of the global miRNA association studies of cognitive trajectory in the discovery and replication datasets. **C** Plot of slope of cognitive trajectory versus individual miR-132 and miR-29a expression in the discovery dataset. Note that the more positive the slope, the more stable the trajectory, and the more negative the slope, the faster the decline.

Each cohort was analyzed separately, and complete results for the discovery (Fig. 2A) and replication cohorts are provided in Supplementary Tables 1, 2. Overall, significance was determined by meta-analysis of both cohorts (Fig. 2B). This yielded six miRNAs associated with the cognitive trajectory in the same direction in both cohorts at adjusted *p* < 0.05 (Supplementary Table 3). miR-132-3p, miR-129-5p, and miR-129-3p had higher expression level in stable cognitive trajectory while miR-29a-3p, miR-99b, and miR-19b had lower expression level in stable cognitive trajectory (Fig. 2C).

Upon examination of correlations among these six miRNAs, we found moderate positive correlations among miR-132-3p, miR-129-5p, and miR-129-3p, with a range of [0.33–0.63] (Supplementary Fig. 1). On the other hand, miR-29a-3p, miR-19b, and miR-99b had small pair-wise correlations between each other and between the rest of the miRNAs, with range of [0.02–0.36] (Supplementary Fig. 1).

### Global miRNA association study of cognitive trajectory adjusting for the eight cerebral pathologies identified 4 significant miRNAs

The six miRNAs found to be associated with cognitive trajectory may act through or independently of the effects of accumulated known cerebral pathologies on the cognitive trajectory. Thus, we simultaneously adjusted for the eight cerebral pathologies (i.e. β-amyloid, neurofibrillary tangles, Lewy bodies, macroinfarct, microinfarcts, cerebral atherosclerosis, cerebral amyloid angiopathy, and hippocampal sclerosis) as well as for potential confounders included in the primary analysis (sex, age, RIN, PMI, study, and the proportions of brain cell types) in our global miRNA association study of cognitive trajectory. We found that only four miRNAs remained significantly associated with cognitive trajectory (miR-132-3p, miR-129-5p, miR-129-3p, and miR-29a-3p), suggesting that they influence cognitive trajectory independently of the eight considered cerebral pathologies (*N* = 438, Supplementary Table 4). Since there were moderate correlations among three of these four miRNAs, we considered all four miRNAs in a single regression model for the outcome of cognitive trajectory to determine the miRNAs most robustly associated with cognitive trajectory. We adjusted simultaneously for the eight pathologies and sex, age, PMI, RIN, study, and proportions of brain cells in this model. We found that only two miRNAs, miR-132-3p and miR-29a-3p, remained significantly associated with cognitive trajectory. Interestingly, miR-132-3p and miR-29a-3p were only mildly inversely correlated with each other (*r* = −0.20, adjusted *p* = 3.22E-05; Supplementary Fig. 1). These results suggest that miR-132-3p and miR-29a-3p contribute to cognitive trajectory independently of the eight cerebral pathologies, and, for this reason, we focused only on miR-132-3p and miR-29a-3p in subsequent experiments.

Interestingly, miR-132-3p was also significantly associated with β-amyloid and tangles, respectively, at adjusted *p* < 0.05 in our earlier miRNA association analysis (Supplementary Tables 5, 6). In secondary analyses, we also performed global miRNA association study of each of the other six measured pathologies but found no miRNAs with an adjusted *p* < 0.05 for any of these traits (Lewy bodies, microinfarct, microinfarct, cerebral atherosclerosis, cerebral amyloid angiopathy, and hippocampal sclerosis). These findings suggest that miR-132-3p influences cognitive trajectory in part through the accumulation of β-amyloid and tangles but also through mechanisms independent of β-amyloid and tangles and other six considered cerebral pathologies.

### Percent variance of cognitive trajectory explained by miR-132-3p and miR-29a

**Fig. 3:**
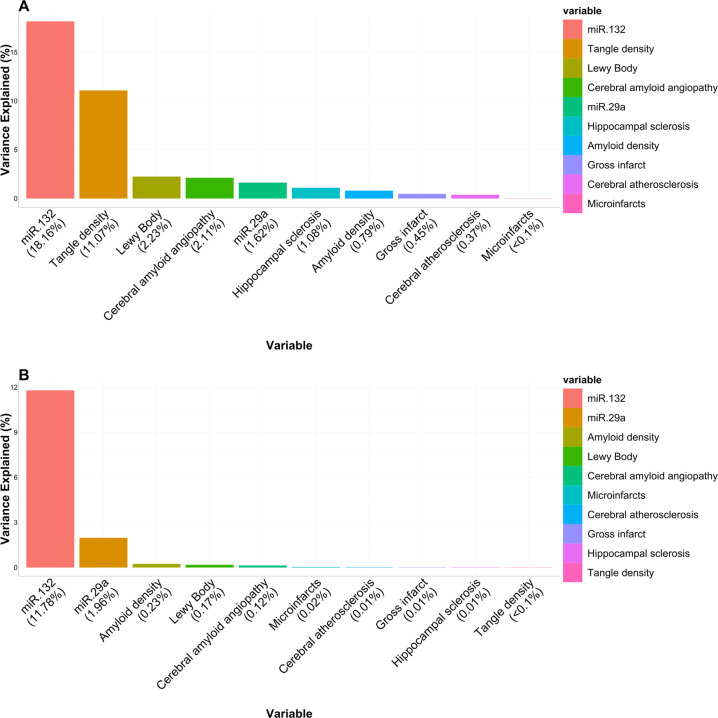
Percent variance of cognitive trajectory explained by miR-132 and miR-29a. This figure shows the percent variance of cognitive trajectory explained by variables in the model. **A** Percent variance of cognitive trajectory explained by miR-132, miR-29a, and each of the eight considered pathologies (amyloid density, tangle density, Lewy bodies, macroinfarct, microinfarcts, atherosclerosis, cerebral amyloid angiopathy, and hippocampal sclerosis), **B** Percent variance of cognitive trajectory explained by miR-132 and miR-29a after effects of the eight cerebral pathologies have been regressed out. For all analyses, the effects of sex, age at death, PMI, RIN, study, and proportions of neurons, astrocytes, oligodendrocytes, and microglia have been regressed out in these percent variance estimates.

We found that miR-132-3p explained 18.2% and miR-29a-3p explained 1.6% of the variance of cognitive trajectory after regressing out effects of sex, age at death, PMI, RIN, study, and the proportions of cell types (Fig. 3). After regressing out the effects of the eight cerebral pathologies, miR-132-3p explained 11.8% and miR-29a-3p 2.0% of the variance of the cognitive trajectory (Fig. 3). These findings underscore the robust effects of miR-132-3p and miR-29a-3p on the cognitive trajectory.

### Relationships between modules of co-expressed genes and miR-132-3p and miR-29a-3p

In the discovery set of ROS/MAP participants, we investigated the relationships among the six cognitive trajectory-associated miRNAs and 47 previously derived modules of co-expressed genes [21]. Both miRNA and mRNA profiles here were generated from the same RNA extracted from the dlPFC [12]. Remarkably, we found that miR-132-3p was associated with 24 of the 47 modules of co-expressed genes with a wide range of correlation coefficients [−0.32 to 0.28] (Supplementary Fig. 2). Importantly, miR-132-3p was associated with all of the top five modules previously found to be most strongly associated with cognitive trajectory (m109, m13, m7, m127, m131; Supplementary Table 7). Notably, the directions of association between miR-132-3p and the modules were consistent with what was expected from the transcriptomic analysis for all of these 24 modules. These findings reflect a broad influence of miR-132-3p on the modules of co-expressed genes and are consistent with the notion that miR-132-3p is a “master regulator” in the aging brain.

miR-29a-3p was significantly associated with 3 modules, m128, m12, and m8, all of which were found to be strongly associated with cognitive trajectory and neurofibrillary tangle burden [21] (Supplementary Table 7).

### Identifying putative targets of miR-132-3p and miR-29a-3p in cognitive trajectory at the transcript level

Since miRNAs alter gene expression through either degrading mRNAs or disrupting the translation of mRNAs into proteins, we sought to identify their targets in the context of cognitive trajectory at the transcript level. To identify transcripts up- or downregulated in cognitive trajectory, we performed a transcriptome-wide differential expression analysis of cognitive trajectory in ROS/MAP participants adjusting for sex, age, PMI, RIN, study, and proportions of cell types. It revealed 1087 transcripts downregulated and 797 transcripts upregulated in cognitive trajectory at adjusted *p* < 0.05 (Supplementary Table 8).

From TargetScan version 7.2 [34], we extracted 474 predicted downstream targets of miR-132-3p and 1256 predicted downstream targets of miR-29a. TargetScan predicts biological targets of miRNAs by searching for the presence of conserved 8mer, 7mer, and 6mer sites that match the seed region of each miRNA [35]. Interaction between a miRNA and their targets in vivo is complex and likely depends on disease state, the abundance of the transcripts, tissue cell type, miRNA abundance, and binding efficacy of the miRNA for a particular transcript compared with other transcripts present in the cell [36, 37].

To identify targets of miR-132-3p and miR-29a-3p in cognitive trajectory at the transcript level, we selected transcripts predicted to be targets of miR-132-3p or miR-29a-3p based on TargetScan [34] and found to be differentially expressed in cognitive trajectory at FDR < 0.05. We identified 64 putative targets of miR-132-3p and 177 putative targets of miR-29a-3p in cognitive trajectory (Supplementary Table 9). Notably these targets belonged to a variety of SpeakEasy derived gene co-expression modules (Supplementary Table 9) suggesting that miR-132-3p and miR-29a-3p target several biological pathways and not just one particular pathway.

### Identify putative targets of miR-132, miR-29a-3p in cognitive trajectory at the protein level

We selected proteins predicted to be targets of these miRNAs based on TargetScan and also found to be differentially expressed in cognitive trajectory at FDR < 0.05. To determine proteins differentially expressed in cognitive trajectory, we used the findings from our meta-analysis of two proteome-wide association studies of cognitive trajectory in Banner and BLSA cohort, respectively (Supplementary Table 10) [28]. These datasets are independent of the ROS/MAP studies and yielded 229 proteins with decreased abundance and 350 proteins with increased abundance in cognitive trajectory at FDR p < 0.05 [28] (Supplementary Table 11). Using the above-described criteria, we found 22 putative targets of miR-132-3p and 41 putative targets of miR-29a-3p in cognitive trajectory at the protein level (Supplementary Table 12).

The overlap between the targets of miR-132-3p at the transcript level (identified in ROS/MAP cohorts) and at the protein level (identified in Banner and BLSA cohorts) were MECP2 and RPH3A. The overlap between the targets of miR-29a-3p in cognitive trajectory at the transcript and protein level included SLC30A3, PDHX, HDGF, and DIRAS1. Furthermore, we found 18 transcripts and 2 proteins that are common targets of both miR-132 and miR-29a in cognitive trajectory (Supplementary Table 13).

In our prior work, we derived protein co-expression modules using Banner proteomic profiles and Weighted Gene Co-Expression Network Analysis (WGCNA) [28]. Here, we found that the putative targets of miR-132-3p belonged to eight different Banner protein co-expression modules (Supplementary Table 12) suggesting that miR-132-3p targets distinct pathways in cognitive trajectory and not necessarily a particular pathway or module of co-expressed proteins. Interestingly, the majority of the putative targets of miR-29a belonged to either M4 protein co-expression module (enriched for myelination), M3 module (enriched for mitochondrial function), M2 module (enriched for catabolic process and apoptosis), or M1 module (enriched for synaptic functions; Supplementary Table 12), suggesting that miR-29a-3p targets primarily proteins involved in myelination, mitochondrial function, catabolic process, apoptosis, and synaptic functions. Moreover, 45% and 41% of putative targets of miR-132-3p and miR-29a, respectively, in cognitive trajectory at the protein level were hub proteins in Banner networks, defined as proteins with intramodular kME of ≥80th percentile (Supplementary Table 12). Hub proteins are likely important proteins because they are highly connected with other proteins in the module and most correlated with the eigenprotein of the module. Hence, more than 40% of the protein targets of miR-132-3p and miR-29a-3p in cognitive trajectory are important proteins in protein co-expression modules.

### In-vitro validation of putative targets of miR-132-3p and miR-29a

**Fig. 4:**
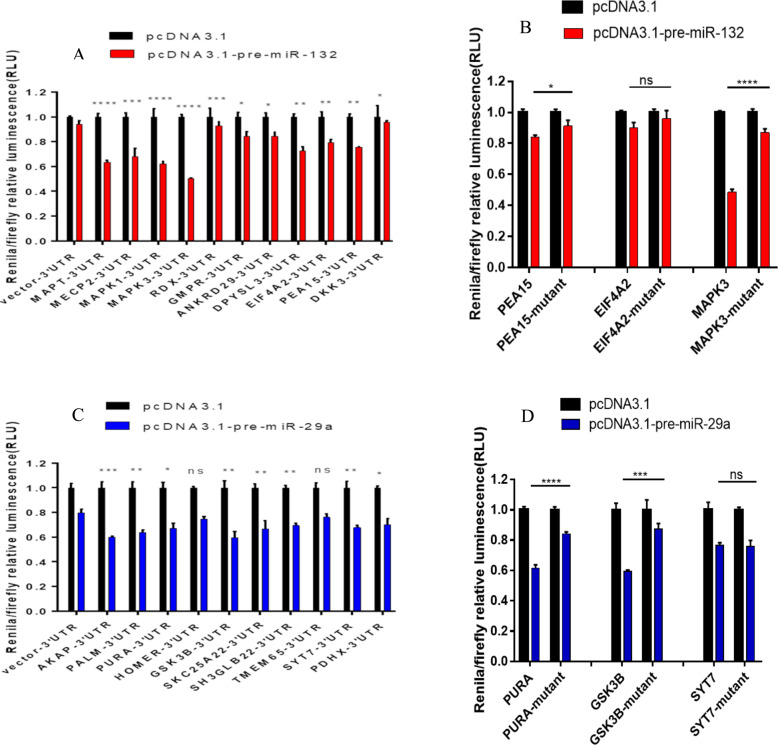
Validation of the targets of miR-132 and miR-29a. This figure shows the results for the Renilla Luciferase assays for the 3’UTR for a selected number of cognitive trajectory-associated genes. **A** Relative R-luc/F-luc ratio in the co-transfection of miR-132 potential targets 3’UTR-reporter constructs with pcDNA3.1-pre-miRNA-132 vs. pcDNA3.1 (as control) in HEK 293 T cells. **B** Rescue experiments to further validate the targets of miR-132 using respective 3’UTR-reporter construct mutant. **C** Relative R-luc/F-luc ratio in the co-transfection of miR-29a potential targets 3’UTR-reporter constructs with pcDNA3.1-pre-miRNA-29a vs. pcDNA3.1 (as control) in HEK 293 T cells (*N* = 3, ns= not significant, *p < 0.05, **p < 0.01, ***p < 0.001, ****p < 0.0001). **D** Rescue experiments to further validate the targets of miR-29a using respective 3’UTR-reporter construct mutant.

We next sought to validate the targets of miR-132 and miR-29a in cognitive trajectory. For validation, we selected genes that met both of the following criteria: (a) identified as targets at the protein level in this study because proteins are the final product of gene expression and because mRNA levels are not highly correlated with protein levels for many genes and (b) with consistent direction of association given miRNA’s canonical action of repressing gene expression. Since miR-132 was downregulated in faster cognitive decline, its downstream targets are expected to be upregulated in faster cognitive decline. For miR-29a, since it was upregulated in faster cognitive decline, its downstream targets were expected to be downregulated in faster cognitive decline. We found 14 and 25 proteins meeting both criteria for miR-132 and miR-29a, respectively. We randomly selected 11 putative targets for miR-132-3p and 10 putative targets for miR-29a-3p for further validation using luciferase reporter assays. We found that the Renila/firefly relative luciferase activity (RLU) of all 11 miR-132-3p putative targets (*MAPT*, *MECP2, MAPK1, MAPK3, RDX, GMPR, ANKRD29, DPYSL3, EIF4A2, PEA15*, and *DKK3)* were significantly decreased in HEK293T cells when their 3’UTR-psiCHECK2 constructs were co-transfected with pcDNA3.1-pre-miR132 compared with pcDNA3.1-sh-scramble (Fig. 4A). Further rescue experiments of the luciferase assay provided evidence that PEA-15 and MAPK3 are direct targets of miR-132 in cognitive trajectory (Fig. 4B).

For the 10 putative targets of miR-29a, the RLU of eight targets (*AKAP5, PALM, PURA, GSK3B, SLC25A22, SH3GLB2, SYT7, PDHX*) were significantly decreased in HEK293T cells when their 3’UTR-psiCHECK2 constructs co-transfected with pcDNA3.1-pre-miR29a were compared with pcDNA3.1-sh-scramble (Fig. 4C). Further rescue experiments suggest that PURA and GSK3B are direct targets of miR-29a in cognitive trajectory (Fig. 4D).

## Discussion

This is the first study to examine the miRNA signature of age-related cognitive trajectories to the best of our knowledge. In particular, we investigated the contribution of miRNAs to the cognitive trajectory in individuals without a diagnosis of dementia at baseline and followed annually for a median of 7 years in a discovery and replication cohort. We found six miRNAs associated with cognitive trajectory after rigorously adjusting for several potential confounding factors. Among these six miRNAs, four (miR-132, miR-29a, miR-129-5p, and miR-129-3p) were associated with cognitive trajectory independently of the eight cerebral pathologies known to contribute to cognitive decline (β-amyloid, neurofibrillary tangles, Lewy bodies, atherosclerosis, gross cerebral infarct, microinfarcts, cerebral amyloid angiopathy, and hippocampal sclerosis). Moreover, among these four, miR-132-3p and miR-29a-3p are two robust contributors to cognitive trajectory because they are the only two that remained significant when we modeled all four miRNAs in a regression model to predict cognitive trajectory.

Together, miR-132-3p and miR-29a-3p capture a striking amount, 19.8%, of the variance of cognitive trajectory. Furthermore, both miR-132-3p and miR-29a-3p are associated with cognitive trajectory independently of eight common cerebral pathologies examined, and they explain 13.7% of the variance in cognitive trajectory once these pathologies have been accounted for. The presence of multiple brain pathologies is common in advanced age [38] and cognitive trajectory likely captures the sum total effect of known and unknown pathologies, in addition to potentially protective biological processes.

Remarkably, miR-132-3p was significantly associated with 24 of the 47 modules of co-expressed genes, including the modules that have the strongest association with cognitive decline, highlighting its broad effects on brain functions in general and on the cognitive trajectory in particular. In a prior study, miR-132 was found to be associated with amyloid and tangles [12]. In this study, we found that miR-132 was also associated with cognitive trajectory even after adjusting for amyloid and tangles. Taken together, miR-132-3p contributes to amyloid and tangles as well as to cognitive trajectory beyond its effects on amyloid and tangles. Indeed, miR-132 contributes to cognitive trajectory beyond its effects on pathologies (i.e., β-amyloid and tangles) and explains 18.2% of the variance of cognitive trajectory. It is not surprising that brain miR-132-3p expression has been found to be associated with Alzheimer’s disease and its pathologies [9, 10, 12, 39] and plasma miR-132 expression was associated with mild cognitive impairment [40]. Consistently, lower expression of miR-132-3p was associated with higher tau burden and tau hyper-phosphorylation, a major hallmark of Alzheimer’s disease [9, 39]. There is also evidence that miR-132 expression in the brain tends to decrease with age [41], plays a direct role in learning [42], regulates neuron morphogenesis [36], and suppresses cortical inflammation [43, 44].

miRNAs, in general, act to repress gene expression by destabilizing their target mRNAs or inhibiting the translation of mRNAs to proteins. We identified predicted direct downstream targets of miR-132-3p and miR-29a-3p in cognitive trajectory at the transcript and protein levels. The target mRNAs identified in this work do not include most genes regulated by MeCP2. It is worth noting that among the 84 identified downstream targets of miR-132-3p in cognitive trajectory, only 2 genes were found to be target at both the transcript and protein levels. Likewise, among the 214 identified targets of miR-29a-3p in cognitive trajectory, only 4 genes were found to be target at both the transcript and protein levels. These findings are consistent with several studies suggesting that mRNA levels do not correlate well with protein levels in many genes, likely due to post-transcriptional, translational, and post-translational regulations [45–47]. Notably 45% and 41% of the downstream targets of miR-132-3p and miR-29a, respectively are hub proteins in independent proteomics dataset implicating these miRNAs and their genes in cognitive decline.

miR-29a was found to be decreased in 11 AD patients with abnormally high BACE1 protein level compared to 21 controls [48], which is opposite to the direction of association between miR-29a and cognitive trajectory in our study of 588 subjects. This could be due to the specific AD subjects with high BACE1 level in that study and may not be generalizable to community-based participants. In line with our miR-132 findings, Hadar and colleagues found lower miR-132 expression in the hippocampus and olfactory bulb in AD subjects compared to controls [49].

Our study has several limitations. First, this is an association study and no causal inference can be made. Second, since the miRNAs were profiled from postmortem brain tissue, the direction of association between miRNA expression levels and cognitive change over time may not reflect their directions of association in real time. Along the same line, while it is generally thought that miRNAs would reduce the expression of their downstream target genes, some of the predicted targets of miR-132 and miR-29a had their mRNA or protein expression levels positive correlated with the levels of miR-132 or miR-29a, respectively. One possible explanation is that we studied miRNAs, mRNAs, and proteins profiled from postmortem brain tissue, which is cross-sectional by nature, and their directions of association in real time cannot be adequately captured. Another possible contributing factor is the dynamic interactions between miRNAs and their target genes as recent work have shown bi-directional interactions between miRNAs and their targets and that targets of miRNAs can influence each other’s expressions [50, 51]. Third, the proteomic dataset was not from the discovery/replication cohort used for the miRNA profiling so the identification of the targets of miR-132 and miR-29a, respectively, at the brain protein level is tentative and needs further validation. Fourth, we did not directly measure the target protein levels in our validation experiments. Fifth, the number of miRNAs detected via the Nanostring platform is lower than would be generated from small RNA-sequencing, highlighting that small RNA-sequencing is the next step to capture more fine-grained effects of miRNAs in cognitive trajectory.

Our study has several strengths. First, we have the largest cohort with postmortem brain miRNA profile and cognitive trajectory. Second, we had a discovery and replication analyses, which reduced false-positive findings. Third, ROS/MAP cognitive performance scores reflect comprehensive cognitive performance because they were the sum of 17 different cognitive tests for each participant. Fourth, we rigorously adjusted for multiple potential confounding factors including the proportions of brain cell types, PMI, and RIN. Fourth, we had eight common cerebral pathologies to adjust for in our analyses.

In summary, we found that miR-132-3p and miR-29a-3p act independently of pathologies to influence 13% of the variance in cognitive trajectory and identified their relevant downstream targets for cognitive trajectory. Our findings lay a foundation for future mechanistic studies to elucidate molecular mechanisms underlying individual variation in cognitive trajectory and develop therapeutics to enhance cognitive resilience in advanced age.

## Supplementary information

Supplementary methods and figures

Supplementary tables
