# Supplementary material for: Brain microRNAs are associated with variation in cognitive trajectory in advanced age
Source: Transl Psychiatry. 2022 Feb 1;12:47. doi: 10.1038/s41398-022-01806-3 (PMC8807720; doi:10.1038/s41398-022-01806-3)
Supplement: Supplementary file 1 — Supplementary methods and figures [file 41398_2022_1806_MOESM1_ESM.docx]

Supplementary Methods and Figures for Wingo et al.

**Supplementary Methods**

***Study design and participants***

Participants for the discovery and replication cohorts for the miRNA analysis were from two longitudinal clinical-pathologic cohort studies of aging and Alzheimer’s disease – the Religious Orders Study (ROS) and Rush Memory and Aging Project (MAP) ^1^. ROS comprises of older Catholic priests, nuns, and monks throughout the USA. MAP recruits older lay persons from the Chicago area. Both studies involve detailed annual cognitive and clinical evaluations and brain autopsy. Participants provided informed written consent, an Anatomic Gift Act for organ donation, and a repository consent to allow their data to be repurposed. The ROS/MAP studies were approved by the Institutional Review Board of Rush University Medical Center. To be included in our miRNA study of cognitive trajectory, participants must be non-demented at baseline in both the discovery and replication cohorts and have at least one follow-up evaluation.

Participants with proteomic profiles were recruited by the Banner Sun Health Research Institute (Banner) and Baltimore Longitudinal Study of Aging (BLSA). Banner Sun Health Research Institute recruits cognitively unimpaired volunteers from the retirement communities of the greater Phoenix, Arizona, USA and subjects with Alzheimer’s disease or Parkinson disease from the community and neurologists’ offices ^2^. These participants were followed longitudinally and annually using standardized general medical, neurological, and neuropsychological tests during life and more than 90% received full pathological examinations after death ^2^. In the current study, we only included subjects that were nondemented at baseline assessment and did not have a diagnosis of Parkinson’s disease. All enrolled subjects in the Banner dataset or their legal representatives sign a Banner Sun Health Research Institute Institutional Review Board-approved informed consent form allowing both clinical assessments during life, several options for brain and/or bodily organ donation after death, and usage of donated biospecimens for approved future research ^2^. The BLSA cohort is a prospective study of aging in community dwelling individuals ^3, 4^. It recruits healthy volunteers aged 20 or older and follow them for life regardless of their changes in health or functional status. Participants were examined at the National Institute of Aging Clinical Research Unit in Baltimore at one to four-year intervals, with more frequent follow-up visits for older participants ^3, 4^. Since BLSA recruited cognitively unimpaired individuals, we included all participants with proteomic data regardless of cognitive score at baseline. The BLSA study was approved by the Institutional Review Board and the National Institute on Aging. Human research at the National Institutes of Health (NIH) is implemented in accord with the U.S. Department of Health and Human Services (45 CFR46) and U.S. Food and Drug Administration (21 CFR 50 and 56) regulations for the protection of human subjects. The NIA IRB is part of the Human Subject Protection Program of the NIH. All BLSA participants provided written informed consent at each visit ^5^.

***Clinical traits and cerebral pathologies***

*Cognitive trajectory* refers to the person-specific rate of change of global cognitive performance over time. Annually, 17 cognitive tests were administered to each ROS/MAP participant. These tests assess the domains of episodic memory, perceptual orientation, perceptual speed, semantic memory, and working memory and have been previously described in detail ^6^. Each year, the raw score from each cognitive test was converted to a Z score using the mean and standard deviation of the cohort at the baseline visit. Then these Z scores were averaged to create the composite annual global cognitive score. We used this composite Z score to represent a participant’s cognitive performance for that follow-up year. Here rate of cognitive change is the random slope from a linear mixed effects model in which the annual global cognitive performance was the longitudinal outcome, follow-up year as the independent variable, adjusting for age at recruitment, sex, and years of education as previously described ^7-10^. The linear mixed model uses a random intercept to allow for the initial cognitive performance to be different among the different subjects and random slope to allow for different rates of change for different subjects. We used the person-specific random slope to represent the rate of change of cognitive performance over time for each subject in our miRNA and transcriptomic analyses. Likewise, for Banner and BLSA participants, person-specific cognitive trajectory was estimated using a linear mixed model. In this model, the annual Mini Mental State Examination (MMSE) ^11^ score was the longitudinal outcome, follow-up year as the independent variable, sex, education, and age at follow-up year as the covariates, and with random intercept and random slope per subject. We used the person-specific random slope to represent rate of change of cognitive performance over time for each subject in our proteomic analyses.

*Cerebral pathologies:* We included eight cerebral pathologies in our ROS/MAP analyses. Neurofibrillary tangles and β-amyloid were identified by molecularly specific immunohistochemistry and quantified by stereology and image analysis, respectively, in eight brain regions – hippocampus, entorhinal cortex, midfrontal cortex, inferior temporal, angular gyrus, calcarine cortex, anterior cingulate cortex, and superior frontal cortex ^10, 12^. Tangle density was determined using systematic sampling, and for each person, tangle density was the average of the tangle densities in eight regions ^10, 12^. β-amyloid score represents the percent area of the cortex occupied by β-amyloid and is calculated by taking the mean of β-amyloid scores in 8 brain regions ^10, 12^. To create approximately normal distribution of tangles and β-amyloid, we took the square root of tangle density and β-amyloid score, respectively.

Lewy bodies were assessed using α-synuclein immunostain and identified as not present, nigral predominant, limbic-type, or neocortical-type as described in detail previously ^8^. Lewy bodies pathology was treated as a dichotomous variable of present versus absent.

Presence of gross cerebral infarct was determined by neuropathologic evaluation blinded to clinical data and reviewed by a board-certified neuropathologist ^13^. Gross cerebral infarct was treated as a dichotomous variable of present or absent. Presence of microinfarcts was determined by neuropathological evaluation blinded to clinical data and reviewed by board-certified neuropathologist in nine regions (midfrontal, middle temporal, entorhinal, hippocampal, inferior parietal, anterior cingulate cortices, anterior basal ganglia, thalamus, and midbrain) and treated as a dichotomous variable ^14^. Large-vessel cerebral atherosclerosis was rated as no significant atherosclerosis observed, mild, moderate, or severe by visual inspection of the vertebral, basilar, posterior cerebral, middle cerebral, and anterior cerebral arteries and their proximal branches ^15^. Cerebral atherosclerosis was treated as a semiquantitative variable.

Cerebral amyloid angiopathy (CAA) was assessed in the midfrontal, midtemporal, angular, and calcarine cortices using immunostain for β-amyloid ^16^. Scores were averaged across these 4 regions ^16^ and treated as a continuous measure for analysis. Lastly, hippocampal sclerosis was evaluated unilaterally in a coronal section of the mid-hippocampus and graded as absent or present based on severe neuronal loss and gliosis in CA1 and/or subiculum ^17^.

*Clinical diagnosis of cognitive status (control, mild cognitive impairment (MCI), or dementia)* was rendered at every assessment based on a three-stage process, including cognitive test scores, clinical judgment by a neuropsychologist, and diagnostic classification by a neurologist, geriatrician, or geriatric nurse practitioner using the criteria set by the joint working group of the National Institute of Neurological and Communicative Disorders and Stroke and the Alzheimer’s Disease and Related Disorders Association (NINCDS/ADRDA) ^18^. After death, a final clinical diagnosis was made considering all clinical data, blinded to all pathologic data.

***microRNA quantification and quality control***

The raw and processed miRNA data from Patrick et al ^19^ were repurposed in the existing work to investigate miRNAs associated with cognitive trajectory independently of cerebral pathologies. miRNA quantification and quality control have been described in ^19^ in detail before. Briefly, total RNA, including miRNA, was extracted from ROS/MAP post-mortem brain tissue from the dorsolateral prefrontal cortex. miRNAs were profiled using the nCounter Human miRNA Expression assay. The miRNAs from the Nanostring RCC files were re-annotated to match the definitions from the miRBase v19 ^19^. We retained miRNAs with call rate ≥ 95% and with an absolute value of > 15 in at least 50% of the samples ^19^. We then removed batch effects using Combat ^19, 20^. After quality control, a total of 292 miRNAs were included in the miRNA association study.

***Transcriptome profiling and quality control***

RNA extracted from ROS/MAP post-mortem dorsolateral prefrontal cortex was sequenced on the Illumina HiSeq with 101-bp paired-end reads using the strand-specific dUTP method with poly-A selection with a coverage of 50 million reads. BAM files were converted to FASTQ format using Picard, followed by alignment of reads to GRCh38 reference genome using STAR ^21^. Gene level counts were computed using STAR ^21^. Genes with < 1 count per million in at least 50% of the samples and with missing length and percent GC content were removed. Additionally, two outlier samples were removed. After quality control, there were 15582 genes to be included in the transcriptome-wide association study of cognitive trajectory.

*Proportions of neurons, astrocytes, oligodendrocytes, and microglia* were estimated from RNA-sequencing data using CIBERSORT ^22^ and cell-type specific signatures from Darmanis et al ^23^. We used the proportions of cell type to adjust for tissue heterogeneity in the global miRNA association study and transcriptome-wide association study of cognitive trajectory.

*SpeakEasy gene co-expression modules*: We used the 47 modules of co-expressed genes created by Mostafavi et al ^10^ using SpeakEasy consensus clustering algorithm on the RNA-sequencing profile described above ^10^. These 47 modules are mutually exclusive modules of co-expressed genes and each module has between 20 and 556 members ^10^. Many of these modules were found to be associated with one or more dementia-related clinical and/or pathological traits ^10^. We examined the association between the cognitive trajectory-associated miRNAs and these 47 modules.

***Proteome quantification and quality control***

Whole-brain proteomes were derived from post-mortem tissue from the dorsolateral prefrontal cortex of Banner and BLSA donors. Proteomic quantification for both cohorts used the approach described in Seyfried et al. ^24^, and a complete description of the methods and all raw proteomic data from the Banner and BLSA cohorts are given in these references ^25, 26^. Briefly, brain-derived tryptic peptides were measured using a NanoAcquity UHPLC (Waters, Milford, FA) and monitored on a Q-Exactive Plus mass spectrometer (ThermoFisher Scientific, San Jose, CA). Raw data were analyzed using MaxQuant v1.5.2.8 with Thermo Foundation 2.0 for RAW file reading capability ^27^. Co-fragmented peptide search was enabled to deconvolute multiplex spectra. The false discovery rate (FDR) for peptide spectral matches, proteins, and site decoy fractions were set to 1%. Protein quantification was estimated by label free quantification (LFQ) algorithm by MaxQuant and only considered razor plus unique peptides for each protein isoform. In essence, this approach estimated the protein abundance for a given protein isoform by using peptides that are unique to the specific isoform and peptides that map to multiple isoforms of the same gene. Only proteins quantified in at least 90% of the samples were included in the analysis. As such, 3710 proteins in Banner and 3933 proteins in BLSA cohorts were included in the cognitive trajectory analysis. Within each cohort, protein abundance was transformed using log2, then batch effects were removed using Combat ^20^, and effects of age at death, sex, and postmortem interval (PMI) were removed using bootstrap regression as described previously ^28^.

***Validation of targets of miR-132 and miR-29a***

Predicted conserved targets of miR-132 and miR-29a were obtained from TargetScan Release 7.1. The 3’ UTR of each putative target was amplified using the cDNA of human RNA and cloned into the psiCHECK2 plasmid (Promega, C8021) following the manufacturer’s recommended protocol. Briefly, cDNA was generated from TRIZOL-isolated total RNA using oligo-dT SuperScript III reverse transcription (Invitrogen, Cat.#1808-093). Primers targeting the 3’UTR incorporated XhoI and PmeI restriction sites with 6bp of flanking sequence, which were used for cloning, and Sanger sequencing was used to confirm the inserted sequence. The 3’-UTR lengths and primer sequences are listed as follows:

List of Oligonucleotides for mir132 potential targets 3’UTR amplification

| Potential targets | FORWARD primers | Reverse primers | Clone site of targets 3’UTR |
| --- | --- | --- | --- |
| ANKRD29 | *GCGGCG*CTCGAGgcatgttcacagattccacagaaac | *GCGGCG*GTTTAAACggaaactcaagattccacagcatg | 249-869 |
| DPYSL3 | *GCGGCG*CTCGAGggcctcagacaccttttaattgc | *GCGGCG*GTTTAAACcaaggaaatcttgtgcagaatcaaagg | 1582-2210 |
| EIF4A2 | *GCGGCG*CTCGAGttcctgggatgagagttttggatg | *GCGGCG*GTTTAAACcaaacttcattaagacatgtgcaatatggc | 1-598 |
| GMPR | *GCGGCG*CTCGAGggaagcgtccaaacctgc | *GCGGCG*GTTTAAACttaagtcttaagataatagcattatattaaagtgaaacc | 32-363 |
| MAPK1 | *GCGGCG*CTCGAGgcgtgtagagcactcaagaaag | *GCGGCG*GTTTAAACgctttgaagtactatgctgaacagttg | 3535-4562 |
| MAPK3 | *GCGGCG*CTCGAGcccagacagacatctctgcacc | *GCGGCG*GTTTAAACcagccatagacacatctctatatttatatattagacggg | 1-635 |
| RDX | *GCGGCG*CTCGAGcggttatagcttactgaagaaatctttccag | *GCGGCG*GTTTAAACctcgcgactgctaataagttataattgg | 1491-2258 |
| MAPT | *GCGGCG*CTCGAGcttttggagctgagatcactc | GCGGCGGTTTAAACtttaatcagagtaataactttatttccaaattcac | 3744-4263 |
| MECP2 | *GCGGCG*CTCGAGgttcagtgtttctgggaga | *GCGGCG*GTTTAAACcctcatgtttggcacaaaagg | 6714-7067 |
| DKK3 | *GCGGCG*CTCGAGgtagggcgagattataaatgaaatttg | *GCGGCG*GTTTAAACagcagttgaagtgatttatgcttg | 1044-1358 |
| PEA15 | *GCGGCG*CTCGAGctctcatgggcctagcatag | *GCGGCG*GTTTAAACgtagttaactttctggcttggg | 552-912 |

List of Oligonucleotides for mir29a potential targets 3′UTR amplification

| Potential targets | FORWARD primers | Reverse primers | Clone site of targets 3’UTR |
| --- | --- | --- | --- |
| AKAP5 | *GCGGCG*CTCGAGgatctgggtgaattcttgcattgtg | *GCGGCG*GTTTAAACcaaaccacagtgaaaaaagtaaaaacctag | 2120-2826 |
| GSK3B | *GCGGCG*CTCGAGgtgtatcattcgtaacccagggag | *GCGGCG*GTTTAAACcagactccacttccgaaccc | 580-1844 |
| HOMER1 | *GCGGCG*CTCGAGgcaatcttttttcctgatattagccaatgg | *GCGGCG*GTTTAAACgtcaatgacgggctgctg | 284-836 |
| PALM | *GCGGCG*CTCGAGggaagtgaggctctatgggg | *GCGGCG*GTTTAAACctgtgagaatgtgcacgaatgtg | 463-1123 |
| PURA | *GCGGCG*CTCGAGgactgagtaggcgttttgtcattattg | *GCGGCG*GTTTAAACctcttcactcaaagatgatagtcatgc | 934-1601 |
| SH3GLB2 | *GCGGCG*CTCGAGgcagccctgccacttaa | *GCGGCG*GTTTAAACcttcatgtccaggaacagcac | 57-850 |
| SLC25A22 | *GCGGCG*CTCGAGcctgagcccagcaccc | *GCGGCG*GTTTAAACcatgacaagacacaaaaagttacaacttc | 5-1425 |
| TMEM65 | *GCGGCG*CTCGAGgcgcttcagaatgcataagtgc | *GCGGCG*GTTTAAACggtcaccatttaacatacatggcaac | 1565-2524 |
| PDHX | *GCGGCG*CTCGAGtcctcaaagataagaagttggtgttcagcttagttg | *GCGGCG*GTTTAAACttggcaagagataacacaatttatttaaatatattccgactcaag | 1-799 |
| SYT7 | *GCGGCG*CTCGAGgcacgggaggtaagggtggtatg | *GCGGCG*GTTTAAACgtttgtggctgttttcctctgtcccttcc | 2426-2999 |

QuikChange Lightning Site-Directed Mutagenesis Kit (Catalog # 210518 ) was used to delete UGGUGCU for miR-29a target site and GACUGUU for miR132 target site. The DNA fragment encoding the pre-miR-132 and pre-miR-29a, respectively, was cloned into pcDNA3.1 and insertions were confirmed by Sanger sequencing. Furthermore, we confirmed there was an increase in the expression levels of mature miR-132 and miR-29a in the HEK-293T cells as shown in Supplementary Figure 3. The cloning method is similar to psiCHECK2-3’UTR construct cloning except for using HindIII and BamHI restriction enzyme to digest PCR products and pcDNA3.1, and using the genomic DNA isolation from HEK293T cells. Twenty nanograms of psiCHECK2-3’UTR constructs of potential targets of miR-132 (wild type/mutant type) together with pcDNA3.1-pre-miR132 or pcDNA3.1-sh-scramble at 500 nanograms were co-transfected into HEK-293T cells (24 wells) with Lipofectamine3000. Likewise, 20 ng of psiCHECK2-3’UTR constructs of potential targets of miR-29a (wild type/mutant type) together with pcDNA3.1-pre-miR29a or pcDNA3.1-sh-scramble at 500 ng were co-transfected into HEK-293T cells with Lipofectamine3000. Then, 48 hours after transfection, luciferase luminescence was revealed with Dual-Luciferase® Reporter Assay System (Promega, E1910) and detected with GloMax ® -96 Microplate Luminometer (Promega). Renilla luminescence was normalized with that of firefly and the signals were presented as renilla/firefly relative luminescence. All transfection assays were performed in triplicates. T-test was used to compare the relative luciferase ratio (ratio of (Relative R-luc/F-luc of 3'UTR reporter constructs + pre-miR-132/Relative R-luc/F-luc of 3'UTR reporter constructs + pcDNA3.1) of each putative targets 3UTR reporter with the vector reporter.

*miRNA Reverse Transcription qPCR:* Total RNA were isolated using TRIzol (invitrogen) according to the manufacture’s recommendations. Reverse transcription (RT) and qPCR were performed using TaqMan MicroRNA RT Kit (4366596) and TaqMan Custom Small RNA Assay Kit. The amplification and melting curve analysis of qRT-PCR reactions were performed with ABI 7500PCR. Results of qRT-PCR were processed based on the 2^(-ΔΔCt) (ΔΔCt target gene = ΔCt target gene - ΔCt reference gene; ΔCt target gene = Ct target gene - Ct internal control).

***Statistical analysis***

*Global microRNA association study of cognitive trajectory*

In the discovery cohort, a global miRNA association study of cognitive trajectory was performed using limma ^29^, adjusting for sex, age at death, RNA integrity number (RIN), post-mortem interval (PMI), study [ROS versus MAP], and proportions of neuron, astrocyte, oligodendrocyte, and microglia. Likewise, a global miRNA association study of cognitive trajectory was performed in the replication cohort as described above except that we could not adjust for proportions of brain cell type. Meta-analysis of the findings from the discovery and replication cohort was performed with METAL using effect size estimates and standard errors ^30^. For all analyses, multiple testing adjustment was addressed with Benjamini-Hochberg (BH) false discovery rate (FDR) ^31^.

*Correlation among the cognitive trajectory-associated miRNAs*

We regressed out effects of sex, age at death, RIN, PMI, study, and proportions of neuron, astrocyte, oligodendrocyte, and microglia from the miRNA profile. We then use this normalized miRNA profile to examine pair-wise correlations among the miRNAs significantly associated with cognitive trajectory using Pearson correlation and adjusting for multiple testing.

*Percent variance of cognitive trajectory*

Likewise, we used the normalized miRNA profile as described above to estimate the percent variance of cognitive trajectory explained by a particular miRNA and each of the eight cerebral pathologies. Specifically, we used a fixed effect model in *variancePartition* package to estimates the effect each of the assessed variables contributes to cognitive trajectory while correcting for the contribution of all the others ^32^. This method considers all the variables jointly and provides a framework for comparing contribution of a particular miRNA to cognitive trajectory to that of each of the known pathologies.

*Correlation between gene co-expression modules and cognitive trajectory-associated miRNAs*

We used the normalized miRNA profile as described above to examine pair-wise correlations between cognitive trajectory-associated miRNAs and each of the 47 modules of co-expressed genes using Spearman correlation, adjusting for multiple testing. Each module was represented by the mean expression level of all the genes assigned to that module.

*Transcriptome-wide differential expression analysis of cognitive trajectory*

The transcriptome-wide differential expression analysis of cognitive trajectory was performed using voom-limma package ^29, 33^. After quality control of the RNA-sequencing data as described above, gene-level counts were transformed into log(CPM) and normalized for library size using trimmed mean of M-values ^34^. Precision weight was calculated using voom to accommodate the mean-variance relationship of RNA-sequencing data. Linear modeling and empirical Bayes moderation were used to perform the transcriptome-wide differential expression analysis of cognitive trajectory adjusting for sex, age at death, study, RIN, PMI, RNA-sequencing batch, proportions of neuron, astrocyte, oligodendrocyte, and microglia. Multiple testing adjustment was addressed with Benjamini-Hochberg (BH) false discovery rate ^31^.

*Proteome-wide association study (PWAS) of cognitive trajectory*

A PWAS of cognitive trajectory was performed in Banner and BLSA, separately, followed by a meta-analysis. These analyses were done previously ^28^. Briefly, in each cohort, a linear regression was performed with cognitive trajectory as the outcome and normalized protein abundance as the predictor. Of note, sex, age at death, and PMI have been regressed from the proteomic profile used in the PWAS. Likewise, sex, age, and education have been regressed during the derivation of cognitive trajectory. A meta-analysis was performed with METAL using effect size estimates and standard errors ^30^. For all analyses, we used Benjamini-Hochberg (BH) method to control the false discovery rate (FDR) ^31^, and declared significantly associated proteins as those with BH FDR p<0.05. We used the proteins found to be associated with cognitive trajectory at FDR <0.05 from the meta-analysis for our integrative miRNA proteomic analysis to identify targets of miR-132 and miR-29a in cognitive trajectory at the protein level.

**Supplementary Figure 1: Correlation among cognitive trajectory-associated miRNAs**

**
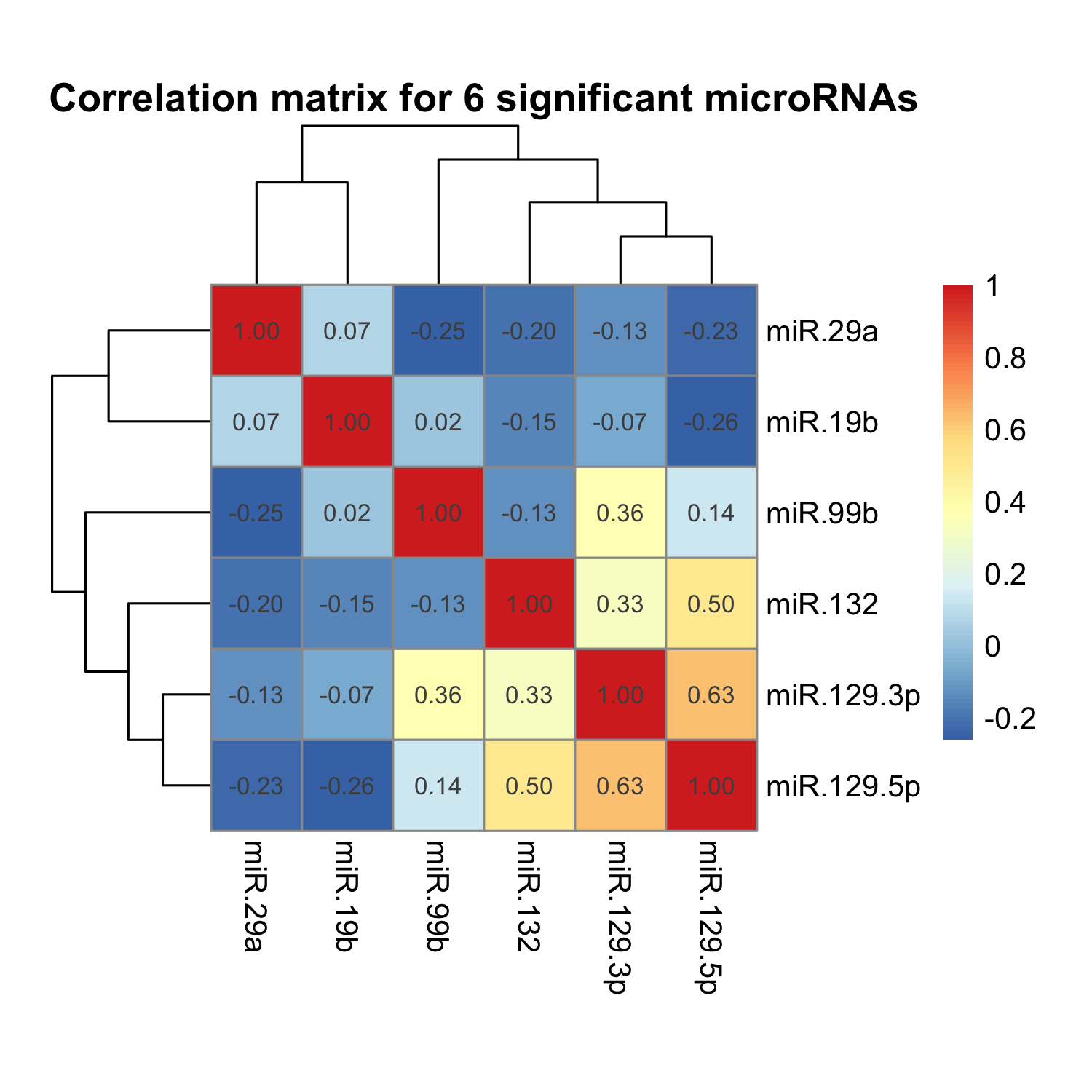
**

This figure shows pair-wise correlations for the six miRNAs significantly associated with cognitive trajectory. All pair-wise correlations were statistically significant at adjusted p<0.05 except for the correlations between miR-19b and miR-29a, miR-19b and miR-129-3p, and miR-19b and miR-99b.

**
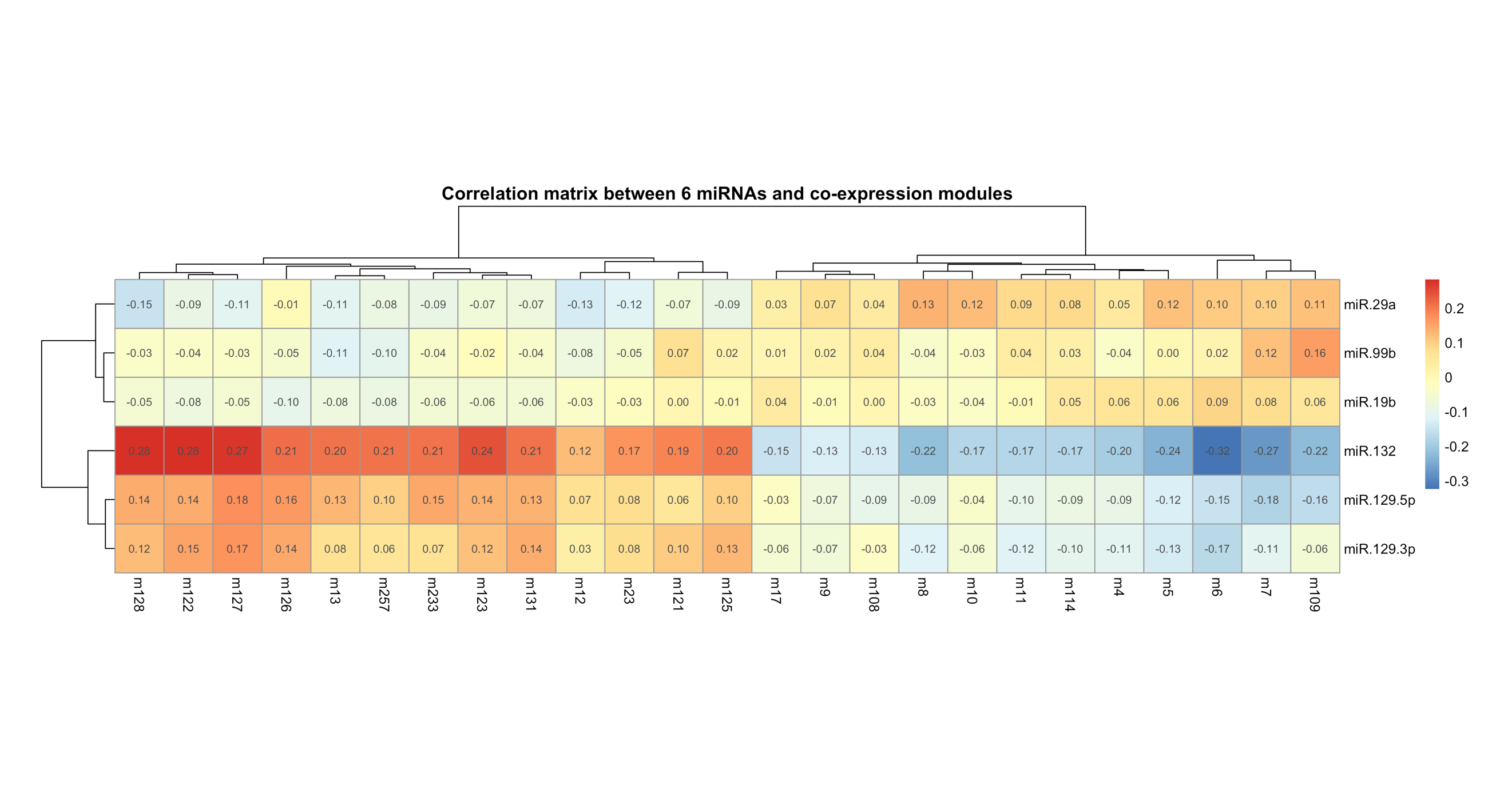
Supplementary Figure 2: Correlation between 6 cognitive trajectory-associated miRNAs and gene co-expression modules.**

This figure shows the pair-wise correlations between the 6 cognitive trajectory-associated miRNAs and 25 gene co-expression modules at adjusted p-value < 0.05 by Spearmen correlation. miR-132 was correlated with 24 modules while miR-29a with 3 modules.

**Supplementary Figure 3**: The relative expression of mature miR-29a and miR-132. (**A)** Mature miR-29a level in the 293T cells transfected with pcDNA3.1-pre-miR-29a, pcDNA3.1, and mock. (**B**) Mature miR-132 level in the 293T cells transfected with pcDNA3.1-pre-miR-132, pcDNA3.1, and mock.


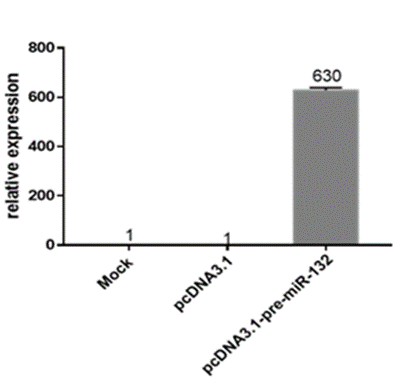

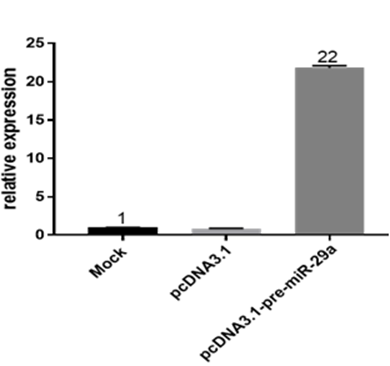


A

B

REFERENCES

1. Bennett DA, Buchman AS, Boyle PA, Barnes LL, Wilson RS, Schneider JA. Religious Orders Study and Rush Memory and Aging Project. *Journal of Alzheimer's disease : JAD* 2018; **64**(s1)**:** S161-s189.

2. Beach TG, Adler CH, Sue LI, Serrano G, Shill HA, Walker DG *et al.* Arizona Study of Aging and Neurodegenerative Disorders and Brain and Body Donation Program. *Neuropathology* 2015; **35**(4)**:** 354-389.

3. Seddighi S, Varma VR, An Y, Varma S, Beason-Held LL, Tanaka T *et al.* SPARCL1 Accelerates Symptom Onset in Alzheimer's Disease and Influences Brain Structure and Function During Aging. *Journal of Alzheimer's disease : JAD* 2018; **61**(1)**:** 401-414.

4. Simpson BN, Kim M, Chuang YF, Beason-Held L, Kitner-Triolo M, Kraut M *et al.* Blood metabolite markers of cognitive performance and brain function in aging. *Journal of cerebral blood flow and metabolism : official journal of the International Society of Cerebral Blood Flow and Metabolism* 2016; **36**(7)**:** 1212-1223.

5. Ferrucci L. The Baltimore Longitudinal Study of Aging (BLSA): a 50-year-long journey and plans for the future. *J Gerontol A Biol Sci Med Sci* 2008; **63**(12)**:** 1416-1419.

6. Wilson RS, Boyle PA, Capuano AW, Shah RC, Hoganson GM, Nag S *et al.* Late-life depression is not associated with dementia-related pathology. *Neuropsychology* 2016; **30**(2)**:** 135-142.

7. De Jager PL, Shulman JM, Chibnik LB, Keenan BT, Raj T, Wilson RS *et al.* A genome-wide scan for common variants affecting the rate of age-related cognitive decline. *Neurobiology of aging* 2012; **33**(5)**:** 1017.e1011-1015.

8. Schneider JA, Arvanitakis Z, Yu L, Boyle PA, Leurgans SE, Bennett DA. Cognitive impairment, decline and fluctuations in older community-dwelling subjects with Lewy bodies. *Brain : a journal of neurology* 2012; **135**(Pt 10)**:** 3005-3014.

9. Wilson RS, Boyle PA, Yu L, Segawa E, Sytsma J, Bennett DA. Conscientiousness, dementia related pathology, and trajectories of cognitive aging. *Psychology and aging* 2015; **30**(1)**:** 74-82.

10. Mostafavi S, Gaiteri C, Sullivan SE, White CC, Tasaki S, Xu J *et al.* A molecular network of the aging human brain provides insights into the pathology and cognitive decline of Alzheimer's disease. *Nature neuroscience* 2018; **21**(6)**:** 811-819.

11. Folstein MF, Folstein SE, McHugh PR. "Mini-mental state". A practical method for grading the cognitive state of patients for the clinician. *J Psychiatr Res* 1975; **12**(3)**:** 189-198.

12. Bennett DA, Wilson RS, Boyle PA, Buchman AS, Schneider JA. Relation of neuropathology to cognition in persons without cognitive impairment. *Ann Neurol* 2012; **72**(4)**:** 599-609.

13. Schneider JA, Wilson RS, Cochran EJ, Bienias JL, Arnold SE, Evans DA *et al.* Relation of cerebral infarctions to dementia and cognitive function in older persons. *Neurology* 2003; **60**(7)**:** 1082-1088.

14. Arvanitakis Z, Leurgans SE, Barnes LL, Bennett DA, Schneider JA. Microinfarct pathology, dementia, and cognitive systems. *Stroke* 2011; **42**(3)**:** 722-727.

15. Arvanitakis Z, Capuano AW, Leurgans SE, Buchman AS, Bennett DA, Schneider JA. The Relationship of Cerebral Vessel Pathology to Brain Microinfarcts. *Brain pathology (Zurich, Switzerland)* 2017; **27**(1)**:** 77-85.

16. Boyle PA, Yu L, Nag S, Leurgans S, Wilson RS, Bennett DA *et al.* Cerebral amyloid angiopathy and cognitive outcomes in community-based older persons. *Neurology* 2015; **85**(22)**:** 1930-1936.

17. Nag S, Yu L, Boyle PA, Leurgans SE, Bennett DA, Schneider JA. TDP-43 pathology in anterior temporal pole cortex in aging and Alzheimer's disease. *Acta neuropathologica communications* 2018; **6**(1)**:** 33.

18. Bennett DA, Wilson RS, Schneider JA, Evans DA, Beckett LA, Aggarwal NT *et al.* Natural history of mild cognitive impairment in older persons. *Neurology* 2002; **59**(2)**:** 198-205.

19. Patrick E, Rajagopal S, Wong HA, McCabe C, Xu J, Tang A *et al.* Dissecting the role of non-coding RNAs in the accumulation of amyloid and tau neuropathologies in Alzheimer's disease. *Molecular neurodegeneration* 2017; **12**(1)**:** 51.

20. Johnson WE, Li C, Rabinovic A. Adjusting batch effects in microarray expression data using empirical Bayes methods. *Biostatistics* 2007; **8**(1)**:** 118-127.

21. Dobin A, Davis CA, Schlesinger F, Drenkow J, Zaleski C, Jha S *et al.* STAR: ultrafast universal RNA-seq aligner. *Bioinformatics (Oxford, England)* 2013; **29**(1)**:** 15-21.

22. Newman AM, Liu CL, Green MR, Gentles AJ, Feng W, Xu Y *et al.* Robust enumeration of cell subsets from tissue expression profiles. *Nature methods* 2015; **12**(5)**:** 453-457.

23. Darmanis S, Sloan SA, Zhang Y, Enge M, Caneda C, Shuer LM *et al.* A survey of human brain transcriptome diversity at the single cell level. *Proceedings of the National Academy of Sciences of the United States of America* 2015; **112**(23)**:** 7285-7290.

24. Seyfried NT, Dammer EB, Swarup V, Nandakumar D, Duong DM, Yin L *et al.* A Multi-network Approach Identifies Protein-Specific Co-expression in Asymptomatic and Symptomatic Alzheimer's Disease. *Cell Syst* 2017; **4**(1)**:** 60-72.e64.

25. The Banner Sun Health Research Institute (Banner) study. <https://www.synapse.org/#!Synapse:syn7170616>, 2017, Accessed Date Accessed 2017 Accessed.

26. The Baltimore Longitudinal Study on Aging (BLSA) study. <https://www.synapse.org/#!Synapse:syn3606086>, 2017, Accessed Date Accessed 2017 Accessed.

27. Cox J, Hein MY, Luber CA, Paron I, Nagaraj N, Mann M. Accurate proteome-wide label-free quantification by delayed normalization and maximal peptide ratio extraction, termed MaxLFQ. *Molecular & cellular proteomics : MCP* 2014; **13**(9)**:** 2513-2526.

28. Wingo AP, Dammer EB, Breen MS, Logsdon BA, Duong DM, Troncosco JC *et al.* Proteome-wide association study of cognitive trajectory reveals enrichment of neuronal mitochondrial proteins in cognitive resilience. *Nature Communications* Accepted 3/12/19.

29. Ritchie ME, Phipson B, Wu D, Hu Y, Law CW, Shi W *et al.* limma powers differential expression analyses for RNA-sequencing and microarray studies. *Nucleic acids research* 2015; **43**(7)**:** e47.

30. Willer CJ, Li Y, Abecasis GR. METAL: fast and efficient meta-analysis of genomewide association scans. *Bioinformatics (Oxford, England)* 2010; **26**(17)**:** 2190-2191.

31. Benjamini Y, Yekutieli D. The Control of the False Discovery Rate in Multiple Testing under Dependency. *The Annals of Statistics* 2001; **29**(4).

32. Hoffman GE, Schadt EE. variancePartition: interpreting drivers of variation in complex gene expression studies. *BMC bioinformatics* 2016; **17**(1)**:** 483.

33. Law CW, Chen Y, Shi W, Smyth GK. voom: Precision weights unlock linear model analysis tools for RNA-seq read counts. *Genome biology* 2014; **15**(2)**:** R29.

34. Robinson MD, Oshlack A. A scaling normalization method for differential expression analysis of RNA-seq data. *Genome biology* 2010; **11**(3)**:** R25.
